# Tomato powder is more effective than lycopene to alleviate exercise-induced lipid peroxidation in well-trained male athletes: randomized, double-blinded cross-over study

**DOI:** 10.1186/s12970-021-00415-7

**Published:** 2021-02-27

**Authors:** Farhad Gholami, Jose Antonio, Cassandra Evans, Khadijeh Cheraghi, Leila Rahmani, Fatemeh Amirnezhad

**Affiliations:** 1 Faculty of Sport Sciences, Shahrood University of Technology, Shahrood, Semnan Iran; 2 Department of Health and Human Performance, Nova Southeastern University, Davie, FL USA; 3 Department of Nutrition, Nova Southeastern University, Davie, FL USA

**Keywords:** Tomatoes, Lipid peroxidation, Antioxidants, Malondialdehyde, Isoprostanes, Oxidative stress

## Abstract

**Background:**

Consumption of nutritional supplements to optimize recovery is gaining popularity among athletes. Tomatoes contain micronutrients and various bioactive components with antioxidant properties. Many of the health benefits of tomatoes have been attributed to lycopene encouraging athletes to consume pure lycopene supplements. The aim of this study was to compare the effect of tomato powder and lycopene supplement on lipid peroxidation induced by exhaustive exercise in well-trained male athletes.

**Methods:**

Eleven well-trained male athletes participated in a randomized, double-blinded, crossover study. Each subject underwent three exhaustive exercise tests after 1-week supplementation of tomato powder (each serving contained 30 mg lycopene, 5.38 mg beta-carotene, 22.32 mg phytoene, 9.84 mg phytofluene), manufactured lycopene supplement (30 mg lycopene), or placebo. Three blood samples (baseline, post-ingestion and post-exercise) were collected to assess total anti-oxidant capacity (TAC) and variables of lipid peroxidation including malondialdehyde (MDA) and 8-isoprostane. Data were analyzed using repeated-measures of ANOVA at *P* < 0.05.

**Results:**

Tomato powder enhanced total antioxidant capacity (12% increase, *P* = 0.04). Exhaustive exercise, regardless of supplement/ placebo, elevated MDA and 8-isoprostane levels (*P* < 0.001). The elevation of 8–isoprostane following exhaustive exercise was lower in the tomato powder treatment compared to the placebo (9% versus 24%, *p* = 0.01). Furthermore, following exhaustive exercise MDA elevated to a lower extent in tomatoe powder treatment compared to the placebo (20% versus 51%, *p* = 0.009). However, such differences were not indicated between lycopene and placebo treatments (*p* > 0.05).

**Conclusion:**

Beneficial effects of tomato powder on antioxidant capacity and exercise-induced lipid peroxidation may be brought about by a synergistic interaction of lycopene with other bioactive nutrients rather than single lycopene.

## Introduction

The production of ROS is a common feature of exercise. It has been established that moderate increases of reactive species is essential to achieving physiologic adaptations of exercise [1]. However, the overproduction of ROS^1^ is likely to result in an imbalance between ROS generation and antioxidant defense, known as oxidative stress. Oxidative stress may damage macromolecules including proteins, lipids, DNA and carbohydrates [1]. Lipid peroxidation, which can be quantified by circulating levels of several biomarkers such as MDA^2^ and 8-isoprostane, can rapidly damage cell membranes and other lipid-containing molecules [1, 2]. Athletes engaging in rigorous training programs, a condition of overproduction of ROS, may be susceptible to oxidative damage.

Athletes may use nutritional strategies to reduce exercise-induced oxidative stress, cell damage, and postexercise fatigue [3]. Incorporating antioxidant-rich foods and supplements in the diet is believed to increase antioxidant capacity and protect the athlete from the harmful effects of oxidative stress [4]. Tomatoes contain micronutrients and various bioactive components with antioxidant properties such as polyphenols, carotenoids, ascorbic acid, a-tocopherol and folate [5]. The aforementioned micronutrients and phytochemicals have been studied for their potential human health benefits, including their ability to counteract oxidative stress [5]. Many of the health benefits of tomatoes have been attributed to lycopene, a carotenoid responsible for the red color found in some fruits [6]. In healthy participants, lycopene has been reported to be a strong singlet oxygen-quencher that can protect macromolecules from oxidative damage [6] and has potential to reduce protein and lipid peroxidation [7]. Daily intake of lycopene (20–40 mg) obtained from tomato products was found to significantly decrease LDL^3^ oxidation [8, 9]. Thus, the results of these studies imply that lycopene be as effective as tomato powder at offsetting the harmful effects of oxidative stress [10]. The recommended daily intake of lycopene is 30–35 mg, which can be obtained from two glasses of tomato juice or from manufactured lycopene supplements in the form of capsules or tablets [11].

Although dietary tomatoes are an abundant source of lycopene, several other nutrients with antioxidant properties are also found in tomatoes. For instance, tomato oleoresin has been suggested to be stronger than lycopene in preventing in-vitro LDL oxidation [12]. It has been reported that supplementation with a moderate dose of a-tocopherol and carotenoids, nutrients present in tomato, over 11 weeks can reduce plasma levels of F_2_-isoprostane in healthy individuals [13]. In an animal study, tomato consumption elicited stronger effects than lycopene administration on prevention of carcinogenesis [14]. Thus, natural tomato may be more potent than single lycopene against oxidative stress and related damages.

Overall, optimizing recovery through natural approaches, such as food and dietary supplements is gaining popularity among athletes. Epidemiological studies have identified numerous human health benefits associated with lycopene intake [15, 16], implying that consuming lycopene or lycopene rich foods could be beneficial in athletic population. To date, no study has evaluated the potential antioxidant benefits of lycopene during exercise or whether a manufactured lycopene supplement can be an applicable alternative to tomatoes products for athletes. Since whole foods contain several bioactive compounds, we hypothesized that these compounds in tomato powder would act in synergy to protect against exercise-induced lipid peroxidation rather than a single compound. Thus, the aim of the present study was to compare the effect of a tomato powder and lycopene supplement on antioxidant capacity and biomarkers of lipid peroxidation in response to exhaustive exercise in well-trained male athletes.

## Methods

### Participants and design

Eleven well-trained males volunteered to participate in the study (Table 1). Participants had training experience of 7.3 ± 1.6 years with 8.7 ± 2.0 h training performed at over 4 sessions per week. Exclusion criteria included smoking, supplement use and medication use at the start of and throughout the study period. The study was approved by the human research ethics committee of The Sport Sciences Research Institute of Iran and complied with the guidelines of the Declaration of *Helsinki*. Written informed consent was obtained from all participants.

**Table 1:** Mean **±** SD of participants’ characteristics and dietary intake

Characteristics	
Age (years)	21.5 ± 1.6
Weight (kg)	64.3 ± 6.5
Height (cm)	174.6 ± 7.6
BMI (kg/ m^2^)	21.0 ± 0.9
Fat mass (%)	12.1 ± 2.6
Skeletal muscle mass (kg)	31.6 ± 4.1
VO_2peak_ (ml/(kg.min))	60.9 ± 6.9
Dietary intake	
Calorie	2532 ± 181
CHO (%)	54.4 ± 2.6
Fat (%)	27.0 ± 2.1
Protein (%)	18.4 ± 3.1

A randomized double-blinded crossover design was used in this study. Upon the first visit, participants attended the laboratory for body composition measurements and baseline assessment of maximal aerobic capacity (VO_2peak_). Body composition analysis was performed using a bioelectrical impedance analyzer (Inbody230, Korea). An incremental exercise test until exhaustion was performed on a motorized treadmill (h/p/cosmos 30000va08, mercury/ med, Germany) to assess VO_2peak_ using indirect calorimetry (MetaMax 3B, Cortex, CPET Germany). Following week long supplementation (tomato powder, lycopene or placebo), participants attended the laboratory to undergo the main exercise tests. The exercise tests were similar at all three conditions. It began with 3 min warm-up at 3 km/h and speed increased to 6 km/h for 3 min and then the speed increased 1 km/h every min until failure. All exercise tests were performed under the similar ambient conditions (24 ± 2 °C) and at the same time of the day from 9 to 11 a.m. A washout period of 2 weeks was set between each treatment.

Participants were provided with an educational handout to help them avoid consuming foods rich in lycopene throughout the study. Lycopene is mainly found in tomatoes, tomato products and some other sources such as watermelon, papayas, grapefruits and guava (Table 2). Participants were asked to refrain from strenuous exercise and consuming caffeine 24 h prior to each exercise trial. The calorie intake and macronutrients consumption were assessed by a 3-day dietary recall (2 weekdays and 1 weekend). Dietary intake of the day preceding the first trial was also assessed using a 24-h recall and participants were asked to replicate the same diet to the best of their ability for subsequent trials. Participants were required to consume the supplement under supervision to ensure compliance.

**Table 2:** Lycopene content of selected foods (μg/ 1 cup) [17]

Tomato products, canned, puree with salt added	54,385
Tomato products, canned, puree without salt added	54,385
Tomato products, canned, sauce, with onions, green papers, and celery	46,135
Tomato juice, canned with salt added	21,960
Tomato juice, canned without salt added	21,960
Vegetable juice cocktail	18,011
Tomatoes, red, ripe, canned, stewed	10,424
Tomatoes, red, ripe, canned, packed in tomato juice	6089
Grapefruit, raw, pink and red, all areas	3264
Papayas, raw	2651

### Blood sample collection and analyses

Blood samples were collected three times during each trial using a catheter inserted into antecubital vein. Following an overnight (8–10 h) fast, baseline blood samples were collected. Then participants received supplements for a week and discontinued supplementation 12 h before main exercise test. Second blood samples were collected following an overnight fast. The third blood samples were collected within 1 min of completion of exercise protocol. All samples were immediately transferred into EDTA containing tubes to separate plasma samples. The tubes were then centrifuged at 4000 r.p.m. at 4 °C for 10 min. The centrifuged samples were pipetted into micro tubes and stored at − 80 °C for later analysis.

MDA was assessed using a quantitative assay on the basis of colorimetric method (ZellBio, Germany) with the intra-assay precision of < 5.8% and inter-assay precision of < 7.6%. TAC was assessed using a quantitative assay on the basis of oxidation-reduction colorimetric assay (ZellBio, Germany). The intra-assay and inter-assay precision was <  3.4 and < 4.2% respectively. A double-antibody sandwich enzyme-linked immunosorbent assay with the intra-assay and inter-assay precision of <  10 and < 12% was used to asses 8_−_isoprostane levels (ZellBio, Germany).

### Supplements

Exercise trials were performed under three different conditions; with tomato powder, lycopene supplement (twenty-first century, USA) or placebo. The rationale for 1-week long supplementation is based on previous studies that showed lycopene is bioavailable with supplementation for 1-week and over [6]. The composition of tomato powder is presented in Table 3. Each serving of tomato powder (60 g) and lycopene supplement contained 30 mg lycopene. Dextrose and 3 g tomato powder were added to the lycopene supplement and placebo to provide all treatments similar in taste. The participants were masked to supplements/ placebo and received them in a cross-over design. The supplements were administered with main dish, to ensure ingestion of lipids such as olive oil at the same time, to enhance the absorption of lycopene [6, 8].

**Table 3:** Tomato powder composition, value per 60 g

Calorie (kcal)	210
Carbohydrate (g)	40.5
Fat (g)	1.96
Protein (g)	9.66
Fiber (g)	5.26
Lycopene (mg)	30
Beta-carotene (mg)	5.38
Phytoene (mg)	22.32
Phytofluene (mg)	9.84
Vitamin C (mg)	76.44
Vitamin E (mg)	3.62
Sodium (mg)	162

### Statistics

Results were expressed as Mean ± SD. The Shapiro-Wilk test was used to confirm the normal distribution of the data. The difference among treatments were determined by a 2-way (treatment x time) analysis of variance for repeated measures. Bonferroni test was used for *post-hoc* analysis where appropriated. The significance level was set to be *P* < 0.05. Data analysis was performed by SPSS for Windows version 25 (SPSS Inc., Chicago, III).

## Results

The compliance to the supplements and placebo consumption was 100% because it was supervised by the research group. The participants did not report any gastrointestinal discomfort or allergic reactions related to the supplementation.

Time-to-exhaustion was 21.12 ± 1.68 min for tomato powder treatment, 20.93 ± 1.54 min for lycopene treatment and 21.19 ± 1.57 min for placebo treatment. There was no significant difference in time-to-exhaustion across three conditions (*p* > 0.05). The analysis of data for variables showed a time x group interaction (F = 5.432, *p* = 0.01, η^2^ = 0.376) and main effects of time (F = 102.151, *p* < 0.001, η^2^ = 0.919) for 8–isoprostane. Regardless of supplement/ placebo, exhaustive exercise elevated 8-isoprostane levels (*P* < 0.001). Follow-up comparisons indicated that 8– isoprostane elevation following exhaustive exercise was lower in the tomato powder treatment compared to the placebo (9% versus 24%, *p* = 0.005), (Table 4). However, such difference was not indicated between lycopene and placebo (*p* > 0.05). Follow-up comparison also did not show significant difference between tomato powder and lycopene treatments (*p* > 0.05). We also observed a time x group interaction (F = 5.071, *p* = 0.001, η^2^ = 0.360) and main effects of time (F = 84.511, *p* < 0.001, η^2^ = 0.904) for MDA. Exhaustive exercise increased MDA concentration regardless of supplement/ placebo (*P* < 0.001). Follow-up comparisons indicated that following exhaustive exercise MDA elevated to a greater extent in tomato powder treatment than the placebo (20% versus 51%, *p* = 0.009), (Table 4). Follow-up comparisons did not show significant difference between lycopene and placebo (*p* > 0.05) and also between tomato powder and lycopene (*p* > 0.05). For TAC, there was a time x group interaction (F = 4.547, *p* = 0.01, η^2^ = 0.336) but main effects of time did not reach significance (F = 1.098, *p* = 0.355, η^2^ = 0.109). The mean values of resting TAC increased following tomato powder ingestion to a greater extent than other treatments (12% increase, *p* = 0.04), (Table 4). There was no significant difference between treatments regarding TAC response to exhaustive exercise (*p* > 0.05).

**Table 4:** Mean **±** SD of variables

	TAC (mM)			MDA (μM)			8-isoprostane (ng/L)		
	Baseline	Post-ingestion	Post-exercise	Baseline	Post-ingestion	Post-exercise	Baseline	Post-ingestion	Post-exercise
**Tomato**	0.25 ± 0.03	0.28 ± 0.02^*#^	0.28 ± 0.02	0.39 ± 0.13	0.36 ± 0.10	0.47 ± 0.11^α#^	277.7 ± 55.5	270.9 ± 60.0	295.9 ± 54.1^α#^
**Lycopene**	0.26 ± 0.04	0.27 ± 0.05	0.27 ± 0.03	0.41 ± 0.07	0.42 ± 0.11	0.57 ± 0.12^α^	269.1 ± 58.3	268.5 ± 59.1	311.4 ± 54.0^α^
**Placebo**	0.28 ± 0.03	0.27 ± 0.02	0.27 ± 0.03	0.40 ± 0.08	0.41 ± 0.07	0.62 ± 0.06^α^	274.4 ± 54.9	275.9 ± 52.3	340.3 ± 54.8^α^

Data presented for all treatments at three time points of before supplementation, after supplementation and following exercise

*Abbreviations*: *MDA* malondialdehyde, *TAC* total anti-oxidant capacity

^*^ Significant difference with baseline values (*P* < 0.05)

^#^ Significant difference with the placebo (*P* < 0.05)

^α^ Significant difference with post-ingestion (*P* < 0.05)

## Discussion

The aim of the study was to compare the effect of tomato powder and lycopene on total antioxidant capacity and lipid peroxidation biomarkers in well-trained athletes. The main finding of the study was that short-term supplementation with tomato powder was more effective than pure lycopene on antioxidant status and lowering exercise-induced biomarkers of lipid peroxidation in well-trained athletes.

Exhaustive exercise increased levels of MDA and 8-isoprostane. These are in accordance with the results of other studies that reported enhanced lipid peroxidation in response to various exercise protocols [18–21]. It has been established that exercise has the potential to enhance free radical generation which may result in acute oxidative stress [2]. The magnitude of oxidative stress and relevant damages depend on various factors such as intensity/ duration of exercise and antioxidant status [2]. Exhaustive exercise can stimulate the overproduction of free radicals. However, a moderate increase in free radical generation by exercise is needed to stimulate certain physiological reactions and adaptations, such as cell signaling and controlling cellular homeostasis [22–24]. This is considered “oxidative strain” rather than a detrimental state of “oxidative stress” [25]. Indeed, it seems to be a double edged sword. Oxidative stress, which is unfavorable to health, can suppress antioxidant defense and can be detrimental to macromolecules. Unsaturated fatty acids, proteins and nucleic acids are the most susceptible cellular components to free radical generation [2]. Due to increases in lipid peroxidation resulting from exercise, protecting the body from oxidative stress damages is crucial to an athlete’s recovery.

We found that 1-week supplementation with tomato powder positively augmented total antioxidant capacity and was more potent when compared to lycopene supplementation. Although this is the first study comparing the antioxidant effects of tomato powder and lycopene supplement following an exercise protocol, our findings are in accordance with the available literature on the effects of tomato and lycopene in health and disease state. For instances, Boileau et al. (2003) indicated that tomato was more effective than lycopene alone in inhibiting carcinogenesis and suggested that antioxidant components other than lycopene exerted a synergistic effect [26]. The body’s total antioxidant capacity is comprised of both endogenous and exogenous, dietary, antioxidants. Consuming micronutrients with antioxidative properties, such as tomatoes products, can support the body’s antioxidant defense system. In addition to lycopene, tomatoes contain carotenoids, a-tocopherol, ascorbic acid and other polyphenols with antioxidant properties that can integrated into the endogenous antioxidant system and exhibit synergistic interaction [27]. The absence of these phenols and other bioactive components in a lycopene supplement, may account for the difference in antioxidant activity. The tomato powder used in this study contained 30 mg lycopene and some other bioactive nutrients including beta-carotene (5.38 mg), phytoene (22.32 mg,) and phytofluene (9.84 mg) as well as vitamin C and E per serving. These nutrients can help increase total antioxidant capacity and synergistically fight against oxidation [27]. The results demonstrated that 1 week of tomato powder supplementation decreased levels of MDA in response to exhaustive exercise but the pure lycopene supplement had no such effect. Alterations in 8-isoprostane following exercise indicated a similar trend to that of MDA. These trends in 8-isoprostane and MDA support the notion that over a short period of time, tomato powder, not synthetic lycopene, has the potential to alleviate exercise-induced lipid peroxidation. MDA is a biomarker of oxidation of total lipid pools but 8-isoprostane belongs to F_2_-isoprostane class and is a reliable biomarker of radical-induced reaction which specifically reflects the oxidation of arachidonic acid [28]. During lipid peroxidation, all lipids especially polyunsaturated lipids are subject to the harmful effects of free radicals [28]. Thus, potential human-health benefits of tomato may be of high importance to athletes. Antioxidant properties found in tomatoes appear to exert a protective effect against lipid peroxidation and the degradation of saturated and polyunsaturated lipids induced by exhaustive exercise.

Regarding effects of tomatoes on lipid peroxidation, Rao and Shen (2002) indicated that 2-week supplementation with tomato products decreased resting serum levels of lipid peroxidation biomarkers in healthy participants [7]. Independently, lycopene and other bioactive components of tomatoes have potential to inhibit lipid peroxidation [5, 6, 27]. We speculate that co-ingestion of these nutrients can favorably boost antioxidant defense to higher levels when compared to lycopene alone. Moreover, some of these nutrients have been reported to exert superior capacity for lipid peroxidation than lycopene. For instance, oleoresin has been reported to be approximately five times more potent than pure lycopene in inhibiting LDL oxidation [12]. It has also been reported that a combination of lycopene and a-tocopherol is more effective than lycopene at inhibiting LDL oxidation [13]. The evidence suggests that lycopene can synergistically act with other components such as oleoresin, a-tocopherol and other nutrients to manifest superior effect. The protective effects of tomato products against oxidative stress cannot be merely attributable to lycopene but rather the synergic effect of lycopene and other antioxidant nutrients.

In contrast to our findings, Sarkar et al. (2012) indicated that 10 weeks of supplementation with both tomatoes and synthetic lycopene following 2 weeks of lycopene restriction improved antioxidant capacity and inhibited lipid peroxidation [10]. They suggested that synthetic form of lycopene was very effective in reducing lipid peroxidation. The discrepancy in our findings and those reported by Sarkar et al. (2012) may be attributed to duration of supplementation and baseline measurements of antioxidant capacity. They applied a 2-week lycopene restricted regimen prior to supplementation which may have lowered initial measurements of lycopene concentration and antioxidant capacity. Moreover, they carried out a longer duration of supplementation which could account for the differences. It may be assumed that longer durations of administration of synthetic lycopene may be more effective to alleviate exercise-induced lipid peroxidation, however this concept has not been explored. Short-duration supplementation with tomatoes and tomatoes products may provide a stronger antioxidative effect in comparison to lycopene. In order to observe the antioxidative effect of lycopene during exercise, longer duration of supplementation may be required. Further research is needed to identify the optimal duration and dose of pure lycopene and its effects on exercise-induced oxidative stress.

### Limitations

The homogenous group of male athlete indicates the effectiveness of tomato powder compared to lycopene for this cohort. Although no sex difference has been reported in the literature regarding benefits of tomatoes and tomatoes products, further studies on female participants may increase our understanding in this area and extend the generalizability of the results. We did not assess the circulatory levels of lycopene before and after supplementation; this may be a limitation to the present study. If we had assessed the concentration of lycopene, we could have determined any association between lycopene levels and biomarkers of peroxidation. However, it may not be a major limitation because it can be concluded from the literature that tomatoes and tomatoes products increase blood levels of lycopene.

## Conclusion

The administration of tomato powder improved antioxidant capacity and alleviated the response of biomarkers of lipid peroxidation to exhaustive exercise in well-trained athletes. Yet, the identical amount of lycopene did not result in similar outcomes. Thus, it may be concluded that beneficial effects of tomato powder on antioxidant capacity and exercise-induced lipid peroxidation might be brought about by the synergistic interaction of lycopene with other bioactive components. This shows that whole tomato contains chemical compounds that can enhance beneficial outcomes in synergy compared to single compound.

## Data Availability

The datasets used and/or analyzed during the current study are available from the corresponding author on reasonable request.

## Abbreviations

MDA: Malondialdehyde
ROS: Reactive oxygen species
LDL: Low-density lipoprotein
TAC: Total anti-oxidant capacity

## Footnotes

1 Reactive oxygen species

2 Malondialdehyde

3 Low-density lipoprotein
